# Comparison of long-term patient reported outcomes of excisional and incisional corporoplasties for Peyronie’s disease

**DOI:** 10.1186/s12610-025-00280-3

**Published:** 2025-09-09

**Authors:** Patrick Daniel Preece, Varun Sahdev, Paul Gerard Davis, Rowland Wyn Rees

**Affiliations:** 1 Department of Urology, University Hospital Southampton, Southampton, UK; 2 Department of Urology, Geelong University Hospital, Geelong, VIC Australia; 3 School of Medicine, Deakin University, Geelong, Australia

**Keywords:** Patient Satisfaction, Peyronie’s Disease, Penile Surgery, Treatment Outcome, Satisfaction des Patients, Maladie de La Peyronie, Chirurgie pénienne, Résultat du Traitement

## Abstract

**Background:**

To compare surgical and long-term patient-reported outcomes (PRO) between excisional (Nesbit) and incisional (Yachia) corporoplasty for correction of uncomplicated Peyronie’s-related penile curvature in a large, single-surgeon cohort. A retrospective audit identified men who underwent Nesbit or Yachia corporoplasty (2015–2021). Operative data was extracted from records. A structured telephone survey captured long-term PRO.

**Results:**

The cohort comprised 101 men (Nesbit = 31, Yachia = 70). Nesbit patients were younger (55 vs 59.7 yr; *p* = 0.02) and had greater baseline curvature (55° vs 45°; *p* = 0.01). Every case was surgically successful (residual curvature < 20° in 100%, < 10° in 58% overall, *p* = 0.66). Yachia procedures were shorter (45 vs 71 min; *p* < 0.05) but required > 2 plications more often (41% vs 7%; *p* = 0.01).

The PRO survey had a 74.3% response rate, with a median follow-up of 5 years. ‘Patient Global Impression of Improvement’ scores reflected strong improvement with median scores of 1 (“very much better”) and 2 (“much better”) in the Nesbit and Yachia groups respectively (*p* = 0.35).

However, this perceived improvement did not translate uniformly into long-term satisfaction. Only 66.7% of respondents reported being “completely” or “mostly” satisfied with the overall outcome (*p* = 0.60). The most frequent cause of dissatisfaction was perceived penile shortening, reported by 85% of men.

Erectile function declined postoperatively in 30% of Yachia and 15% of Nesbit patients (*p* = 0.03), though this is possibly confounded by the older age of the Yachia cohort. Bothersome curvature recurrence and post-operative cosmesis did not significantly impact satisfaction in either group.

**Conclusions:**

Both Nesbit and Yachia corporoplasties provide effective and durable results with comparable long-term patient satisfaction. Regardless of technique, subjective reporting of penile shortening was particularly pervasive and was highly bothersome. This underscores the need for meticulous pre-operative counselling.

**Supplementary Information:**

The online version contains supplementary material available at 10.1186/s12610-025-00280-3.

## Résumé

**Contexte:**

Comparer les résultats chirurgicaux et à long terme rapportés par les patients entre la corporoplastie excisionnelle (Nesbit) et incisionnelle (Yachia) pour la correction de la courbure pénienne non compliquée liée à la maladie de La Peyronie, dans une grande cohorte d’un unique chirurgien. Un audit rétrospectif a permis d’identifier des hommes ayant subi une corporoplastie de Nesbit ou de Yachia (2015-2021). Les données opérationnelles ont été extraites des dossiers. Un sondage téléphonique structuré a permis de recueillir les résultats à long terme rapportés par les patients.

**Résultats:**

La cohorte comprenait 101 hommes (Nesbit = 31, Yachia = 70). Les patients traités par Nesbit étaient plus jeunes (55 ans versus 59,7 ans ; *p* = 0,02) et avaient une courbure de base plus importante (55° versus 45° ; *p* = 0,01). Tous les cas ont été couronnés de succès (courbure résiduelle <20° pour 100 %, et <10° sur 58 % globalement, *p* = 0,66). Les procédures de Yachia étaient d’une durée plus courtes (45 vs 71 min ; *p* < 0,05) mais nécessitaient plus souvent plus de 2 plicatures (41 % vs 7 %; *p* = 0,01).

L’enquête à long terme sur les résultats rapportés par les patients a eu un taux de réponse de 74,3 %, avec un suivi médian de 5 ans. Les scores d’ « Impression globale d’amélioration du patient » ont montré une forte amélioration, avec des scores médians de 1 (« vraiment mieux ») et de 2 (« beaucoup mieux ») dans les groupes Nesbit et Yachia respectivement (*p* = 0,35). Cependant, cette amélioration perçue ne s’est pas traduite uniformément par une satisfaction à long terme. Seuls 66,7 % des répondants ont déclaré être « entièrement » ou « en grande partie » satisfaits du résultat global (*p* = 0,60). La cause d’insatisfaction la plus fréquente était la perception d’un raccourcissement du pénis, déclarée par 85 % des hommes.

En postopératoire, la fonction érectile a diminué chez 30 % des patients Yachia et 15 % des patients Nesbit (*p* = 0,03), bien que cela puisse être dû à l’âge plus avancé dans la cohorte Yachia. La récurrence gênante de la courbure, et l’esthétique postopératoire, n’ont pas eu d’impact significatif sur la satisfaction dans l’un ou l’autre des groupes.

**Conclusions:**

Les corporoplasties Nesbit et Yachia offrent toutes deux des résultats efficaces et durables, avec une satisfaction comparable à long terme pour les patients. Quelle que soit la technique, les rapports subjectifs sur le raccourcissement du pénis étaient omniprésents et très gênants. Cela souligne la nécessité de conseils préopératoires méticuleux.

## Introduction

Peyronie’s disease is a common and distressing condition that can significantly compromise a man’s sex life and negatively impact his self-esteem. Up to one-third of patients eventually undergo surgery to alleviate the disease's effects [1, 2].

Men might be considered for penile implant insertion or incision and grafting procedure if they develop a complex deformity, defined by features including extreme curvature, buckling from wasting or hinge defects, distal flaccidity, severe erectile dysfunction or short stretched penile length. Otherwise, uncomplicated curvatures are commonly treated with a corporoplasty procedure [3].

Whilst several techniques exist, they all employ the same principle of shortening the tunica albuginea of the (uninvolved) convex side of the penile shaft, to lever straight the (disease affected) convex side and restore symmetry.

The Nesbit procedure is an excisional corporoplasty, whereby an ellipse of tunica is excised and closed. First described in 1965 for congenital penile curvature [4], the procedure was adapted to treat Peyronie’s disease by Pryor and Fitzpatrick in 1979 [5]. In comparison, the Yachia procedure is an incisional corporoplasty. It relies on the Heineke-Mikulicz principle of closing a longitudinal incision of the tunica transversely. First described in 1984 by Lemberger et al. [6], the technique was refined and popularised by Yachia to achieve a smoother finish of the corpora [7].

Whilst there are proponents of both techniques, there is to date a paucity of comparative research. Although many single arm cohort studies exist, it is difficult to compare Nesbit and Yachia outcomes due to the lack of standardised reporting and the heterogeneity of the definition of ‘success’. Results typically focus on objective measures such as improvement in curvature. Subjective Patient Reported Outcomes (PRO) are less commonly utilised, although arguably give a more holistic appraisal of outcomes through measures of patient satisfaction and experience of post-operative changes such as penile shortening. It is hypothesised that the differing mechanical approaches of incisional and excisional corporoplasty may yield distinct long-term PRO. As such, this study aims to evaluate the PRO of a large, single surgeon cohort of Peyronie’s patients undergoing surgical correction with either Nesbit or Yachia corporoplasty.

## Patients and methods

A retrospective analysis of surgical outcomes was performed in conjunction with a telephone survey to assess long-term PRO within a large, single surgeon series of Nesbit and Yachia corporoplasties. Men who underwent surgery for acquired penile curvature (i.e. Peyronie’s disease) between the years of 2015–2021 were included. This study period was chosen as the surgical technique utilised by the high-volume reconstructive urologist was unchanged over this time. Patients with congenital curvature were excluded. All men had at least a 6-month history of stable plaque. Curvature was assessed pre-operatively with intra-cavernosal injection in 33% of patients; the remainder via patient-provided photography.

### Surgical technique

Both Nesbit and Yachia procedures were preferentially performed through a degloving subcoronal incision. Uncircumcised men were recommended to have a concomitant circumcision. A saline artificial erection test was performed using a high-flow fluid dispensing system [8] to demonstrate both location and severity of curvature. Allis forceps were applied to the tunica to decide the optimal site for corporoplasty, before creating a window through Buck’s fascia to perform either of the operative techniques.

**Fig. 1:**
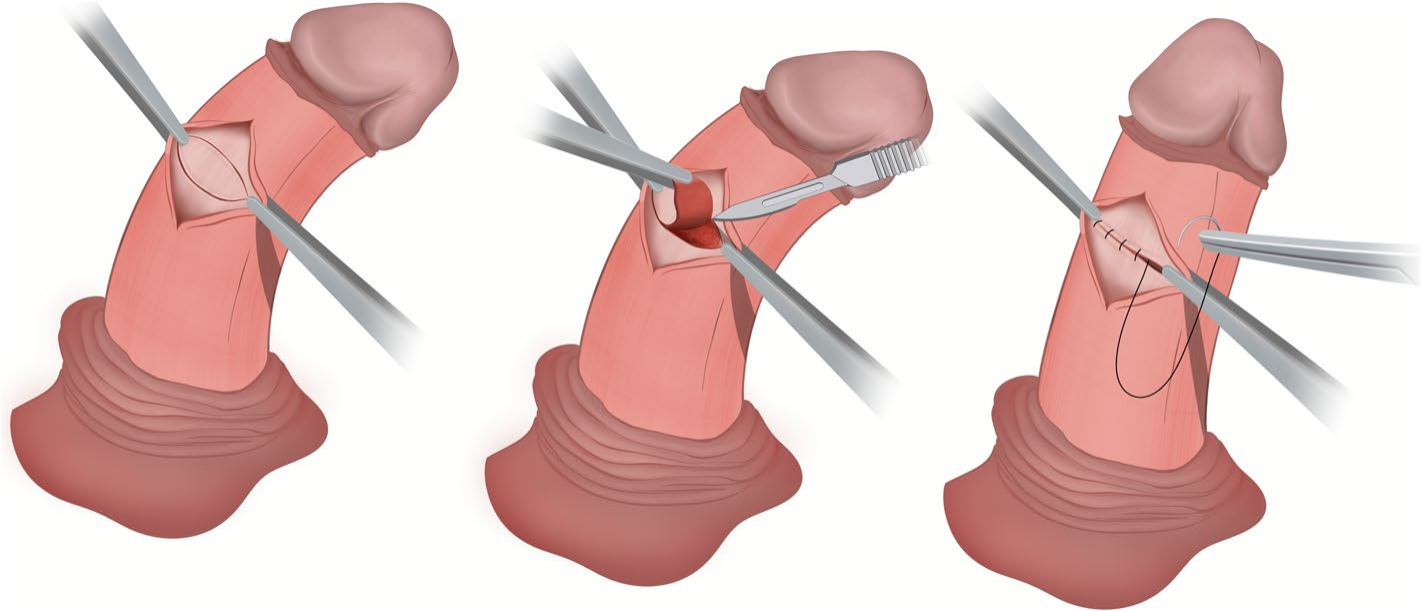
Nesbit corporoplasty- key steps. Create a window in Buck’s fascia, excise a small ellipse of tunica albuginea and close with 0 polydioxanone

The Nesbit procedure involved a traditional full thickness, transverse elliptical excision of the tunica albuginea. The corporotomy was closed using buried, interrupted sutures of slow dissolving 0 polydioxanone (Fig. 1).

**Fig. 2:**
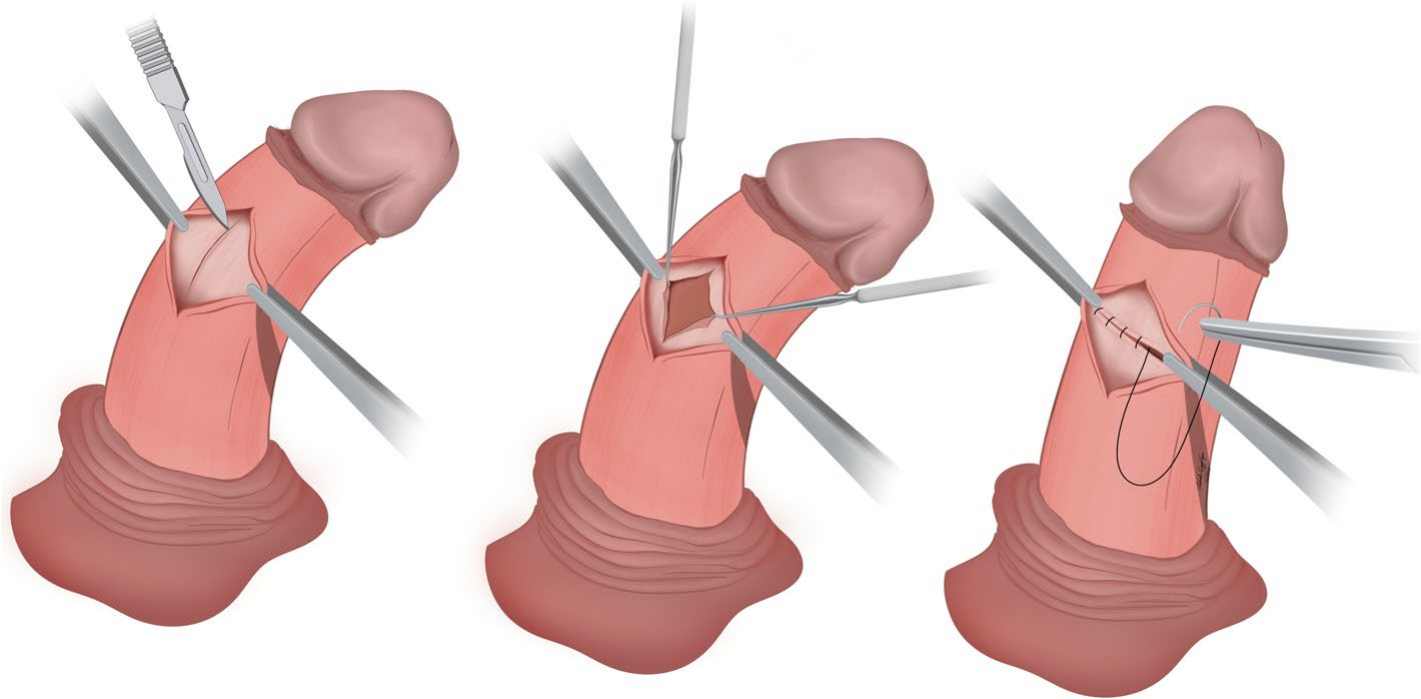
Yachia corporoplasty- key steps. Create a window in Buck’s fascia, longitudinally incise tunica albuginea and close transversely with 0 polydioxanone

In contrast, for the Yachia procedure, a short longitudinal incision up to 1 cm in length, was made full thickness through the tunica albuginea. It was subsequently closed in a transverse fashion also using buried, interrupted sutures of 0 polydioxanone. ‘Dog ears’ at the wound edges were buried with absorbable 4/0 polyglactin (Fig. 2).

Following either technique, a repeat artificial erection test was performed to assess for any further required correction, prior to completing a layered closure of Buck’s, dartos and skin, and applying a pressure bandage dressing.

### Patient assessment

Retrospective data was collected regarding patient and disease characteristics, as well as objective surgical outcomes including residual curvature. To assess long-term PRO following surgery, all men were invited to complete a questionnaire, administered over the phone by a single researcher. This audit tool was developed specifically for this study to evaluate domains relevant to post-operative satisfaction and complications. It incorporated the ‘Patient Global Impression of Improvement’ (PGI-I); a single item instrument that asks patients to rate their condition now compared to before treatment on a 7-point scale from 1(“Very much better”) to 7 (“Very much worse”) [9]. Additional questions utilised unvalidated Likert scales (1–5) to assess specific domains: bother related to recurrent curvature, perceived penile shortening, altered penile sensation, erectile dysfunction and cosmesis. These items were designed to capture patient-perceived outcomes in a structured and reproducible format. The complete questionnaire is provided as supplementary material.

### Statistical analyses

Univariate analysis was performed using the software package STATA (StataCorp, College Station, TX, USA). Non-parametric statistical tests were used, including Fisher’s exact test for categorical data and Wilcoxon rank sum for continuous data and Likert scales.

## Results

A total of 101 men underwent Nesbit (30.7%) or Yachia plication (69.3%) between 2015–21 (Table 1).

**Table 1:** Patient and surgery characteristics

	**Nesbit**	**Yachia**	***p* value**
**Number of patients**	31	70	
**Median age, yrs (range)**	55 (20.1–70.8)	59.7 (37.1–75.5)	**0.02**
**Median curvature ° (range)**	50 (30–90)	45 (20–90)	**0.01**
**Curvature correction (%)**			0.66
< **10°**	61	54.5	
**10–20°**	39	45.5	
> **20°**	0	0	
**Plications required (%)**			**0.01**
**1**	41.9	18.6	
**2**	51.6	40	
> **3**	6.5	41.4	
**Uncircumcised pre-op (%)**	58.6	69.7	0.21
**Circumcised intra-op (%)**	68.8	58.7	0.29
**Adjunct manoeuvres**			0.37
**Dorsal vein stripping**	0	2 (2.9%)	
**Neurovascular bundle mobilisation**	1 (3.2%)	0	
**Pre-op Erectile dysfunction (%)**	35	48	0.27

Fisher’s exact test, Wilcoxon rank sum

The Nesbit approach was favoured in younger men (median 55 vs 59.7 years, *p* = 0.02), with more severe curvatures (55 vs 45 degrees, *p* = 0.01). Nonetheless, all were successfully improved to < 20 degrees, with < 10 degrees achieved in 61% of the Nesbit and 55% of the Yachia group (*p* = 0.66). The ability to successfully correct the curvature with a single correction favoured Nesbit over Yachia (42% vs 19% (*p* = 0.01)). 41% of the Yachia group required 3 or more plications to achieve satisfactory straightening. Duration of procedure favoured the Yachia procedure, with a median operative time of 45 versus 71 min for the Nesbit procedure (*p* < 0.05).

The majority (80%) of curvatures were dorsal or dorsolateral. 17% were lateral and only 3% had a ventral component. 3% of cases underwent concurrent dorsal vein stripping or neurovascular bundle mobilisation. There was no significant difference between Nesbit and Yachia groups regarding intra-operative circumcision rates (*p* = 0.29) nor pre-existing erectile dysfunction (*p* = 0.27).

Post-operative complication rates were low: 2% developed a haematoma, 33.3% of uncircumcised men that declined concomitant circumcision developed subsequent phimosis.

The response rate of the PRO survey was 74.3%, with a median interval of 5 years (range 0.6–7.1) from surgery (Table 2).

**Table 2:** Long-term patient reported outcomes

	**Nesbit**	**Yachia**	***p* value**
**Patient Global Impression of Improvement (PGI-I)**	1	2	0.35
*1* = *very much better, 7* = *very much worse (Median)*			
**Overall satisfaction**	4	4	0.60
*1* = *Dissatisfied, 5* = *Completely satisfied (Median)*			
**Recurrent Curvature**			
Self-reported Recurrent Curvature (%)	20	24.1	0.49
Degree of bother if yes *(1* = *Extremely, 5* = *Not at all; Median)*	2	4	0.55
**Loss of length**			
Self-reported shortening (%)	85	85.5	0.61
Degree of bother if yes *(1* = *Extremely, 5* = *Not at all; Median)*	4	4	0.87
**Cosmesis (post-op penile appearance)**			
Degree of bother *(1* = *Extremely, 5* = *Not at all; Median)*	5	5	0.77
**Penile hypoaesthesia**			
Self-reported decrease in sensitivity (%)	15	14.5	0.61
Degree of bother *(1* = *Extremely, 5* = *Not at all; Median)*	2	4	0.39
**Erectile function**			
Quality of erections *(1* = *Much worse, 5* = *Much Better; Median)*	3	3	**0.03**

Likert Scales, Wilcoxon rank sum

### PGI-I

**Fig. 3:**
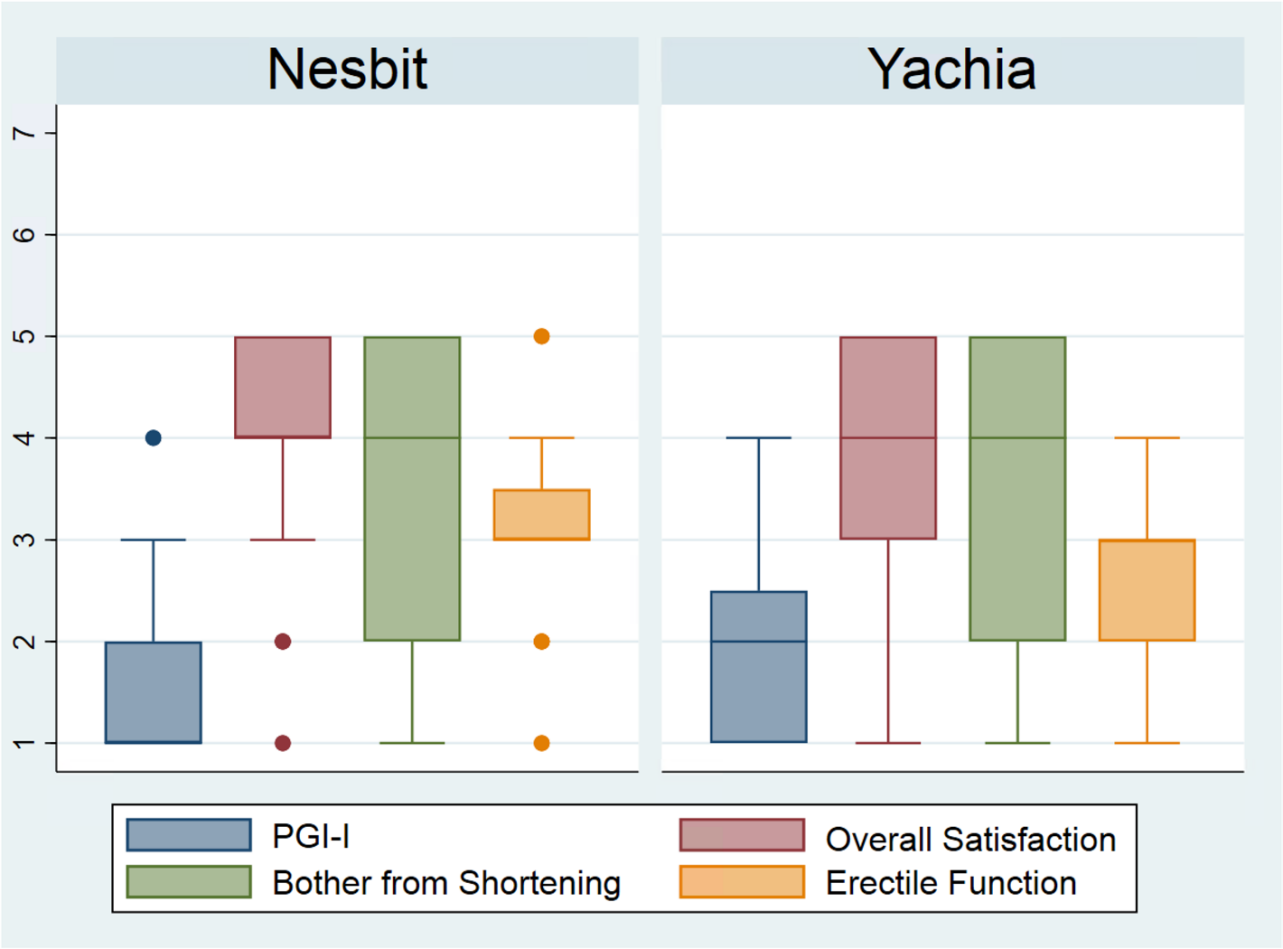
Likert scale box plots- Nesbit vs Yachia. PGI-I: *1* = *Very much better, 2* = *Much better, 3* = *A little better, 4* = *No change, 5* = *A little worse, 6* = *Much worse, 7* = *Very much worse*. Overall Satisfaction: *1* = *Dissatisfied, 2* = *Somewhat dissatisfied, 3* = *Neither satisfied nor dissatisfied, 4* = *Somewhat satisfied, 5* = *Completely satisfied*. Bother from Shortening: *1* = *Extremely bothered, 2* = *Very bothered, 3* = *Moderately bothered, 4* = *A little bothered, 5* = *Not at all bothered*. Erectile Function: 1 = Much worse, 2 = Slightly worse, 3 = No difference, 4 = Slightly better, 5 = Much better

Both Nesbit and Yachia cohorts scored highly (median value of 1 vs 2, *p* = 0.35) with the single question PGI-I, rating their condition from 1- ‘Very much better’ to 7- ‘Very much worse’ compared to before surgery (Fig. 3).

### Overall satisfaction

80% of Nesbit and 62% of Yachia patients were either “completely” or “mostly satisfied”, whereas 15% Nesbit and 20% Yachia were either “dissatisfied” or “somewhat dissatisfied” with the overall outcome (*p* = 0.60).

10% of Nesbit and 11% of Yachia patients regretted having surgery. The most cited reason for dissatisfaction was penile shortening. 30.1% of men surveyed never had intercourse at any stage following surgery.

### Recurrent curvature

20% of Nesbit and 24.1% of Yachia patients reported a recurrent curvature (*p* = 0.49), although only 9.5% found this subsequently interfered with intercourse.

### Loss of erect length

85% of each cohort believed surgery caused shortening, with 36% of Nesbit and 34% of Yachia patients either “extremely” or “very bothered” by this perceived loss of length (*p* = 0.92). 69.1% believed they had lost in excess of 2 cm of length as a consequence of the surgery.

### Penile cosmesis

93% of Nesbit and 94% of Yachia patients reported they were ‘not at all bothered’ regarding the cosmetic outcome and appearance of their penis (*p* = 0.77).

### Penile hypoaesthesia

There was no difference in rates of decreased sensation (15% vs 14.5%, *p* = 0.61) between Nesbit and Yachia cohorts. However, altered glans sensitivity was reported in 28.1% of men who underwent concomitant circumcision vs 4.8% of those who didn’t (*p* = 0.01).

### Erectile function

A significant difference was noted in PRO post-operative erectile function (*p* = 0.03). 25% of Nesbit and 4% of Yachia patients reported their erections to be improved following surgery, with 60% vs 67% reporting no change. 15% of Nesbit and 30% of Yachia patients found their erections to be worse as a consequence of surgery; all were successfully managed with medications, intra-cavernosal injections or vacuum erection device.

## Discussion

### Patient reported outcomes

Both Yachia and Nesbit proved effective techniques for correcting penile curvatures, with 100% patients rendered functionally straight (less than 20-degree residual curvature) and 56.7% improved to less than 10 degrees. Yet this study’s findings suggest that correction of a Peyronie’s curvature alone is a poor metric for long-term patient satisfaction. Despite all cases being considered a technical ‘success’, long-term PRO (with a 5-year median follow up) found that only 66.7% remained satisfied overall.

10.7% of patients regretted undergoing a straightening procedure, with penile shortening being the most frequently cited reason for dissatisfaction. 85.3% of both cohorts reported a subjective loss of length, with 34.4% reporting “very” or “extreme bother” because of it.

Whilst validated questionnaires are favoured research tools because of their standardised metrics, those currently available for Peyronie’s disease have limited utility. The International Index of Erectile Function (IIEF) and Peyronie’s Disease Questionnaire (PDQ) presume patients have engaged in recent sexual activity which is often not the case. They also fail to assess several domains which are notionally important regarding post-operative outcomes. By creating a bespoke questionnaire, this study was able to quantify the subjective judgment of Peyronie’s patients regarding how surgery had altered penile length, appearance, sensation, erectile function and recurrent curvature; domains that are important to overall quality of life but are insufficiently assessed with available validated tools. The inclusion of the PGI-I provided a pragmatic and broadly accepted single-item anchor for perceived change. Originally developed for assessing urinary symptom response, the PGI-I has since been widely adopted across a range of conditions, including Peyronie’s disease [10].

### Loss of length

Only one other publication has directly compared PRO between Nesbit and Yachia cohorts for Peyronie’s disease [11]. They reported similar overall satisfaction rates (“satisfied” or “very satisfied”) of 83% (Nesbit) and 79% (Yachia), *p* > 0.05, with a shorter median follow up of 22 and 12 months respectively. In contrast to our results, they found a statistical difference in penile shortening as a PRO, which affected 67% of Yachia and 37% Nesbit patients (*p* = 0.01). The degree of bother was not quantified, and the severity of pre-operative curvature was not stated between the groups, nor the number of plications utilised in each case.

Assessing PRO for a combined Yachia/Nesbit group that included both Peyronie’s and congenital curvature patients, Baldini et al. [12] found a dissatisfaction rate of 25%. This was again largely attributed to 72.5% of respondents describing a “clear” length loss from the surgery, with an estimated mean of 2.4 cm. Similarly, 69.1% of men in our study believed they had lost more than 2 cm.

In contrast, in one of the largest retrospective Peyronie’s cohort studies to date, Ralph et al. [13], reported shortening of > 2 cm in only 4.7%, compared to < 1 cm in 86.6% of 359 men undergoing Nesbit. Such disparity in results reflects the difference in ‘objective’ chart review compared to ‘subjective’ PRO. Nonetheless, acknowledging that an overestimation of lost length may result from patient bias, does not lessen the impact it has on overall satisfaction rates.

### Corporoplasty techniques

Although Yachia and Nesbit are both widely practiced corporoplasty techniques, corporal plication is also favoured by many (e.g. The Essed & Schroeder [14] and the ‘16 dot’ procedures [15]). These non-excisional imbrication techniques achieve a straight erection in a similar fashion by shortening the convex side of the corpora. Studies suggest outcomes may be comparable (although not superior) to Nesbit and Yachia [16]. Corporal plication has been shown to be a faster operation and is generally thought to have a shorter learning curve [17]. Nevertheless, plication techniques that rely solely on suture material maintaining its integrity, experience higher rates of certain complications. These include discomfort from palpable knots and early curvature recurrence from suture failure [18]. Such complications are infrequent with the Yachia and Nesbit approaches due to the inherent strength of tunical tissue healing. Only 1.3% of study participants were bothered by the cosmetic outcome of the surgery including the presence of palpable sutures.

### Erectile dysfunction

The only domain in our PRO questionnaire that demonstrated a statistically significant difference between Nesbit and Yachia groups was erectile function. Impaired erectile function (“worse” or “much worse”) was reported twice as often by the Yachia cohort (30% vs 15%, *p* = 0.03). However, the cause of this discrepancy remains unclear. On average, the Yachia procedure required a higher number of corporoplasties to achieve a straight erection. 41.4% of Yachia vs. 6.5% of Nesbit patients required 3 or more separate plications (*p* = 0.01). It is plausible that added corporal incisions compound any damage to underlying cavernosal tissue. However, the Yachia cohort was significantly older (*p* = 0.02) which would typically signify a higher prevalence of ED, especially given the long-term median follow up of 5 years. In comparison, Licht et al. [11] with a shorter follow up period found low (< 4%) de-novo ED rates in both Yachia and Nesbit groups. Kadioglu et al. [19] found there was a high overlapping incidence of vascular disease risk factors in Peyronie’s patients, and that ED was progressive over time.

This study found that almost a third of men surveyed never had intercourse at any stage following surgery. Paez et al. [20] similarly reported, that with a median follow up of 5.9 years, 52.3% of men following a plication procedure for Peyronie’s did not resume an active sex life. Gamidov et al. [21] found a forced or voluntary abstinence rate of 16.7% with median follow up of 9.5 years. The reasons are myriad (difficulties with erections, waning libido, prostate cancer treatment, pre-existing relationship status and relationship difficulties). Some patients may be addressing their penile curvature in a hope to salvage a relationship or start a new one. This study therefore serves as a caution that correcting the curvature may not necessarily lead to the resumption of an active sex life.

### Limitations of the study

This study has several limitations inherent to its retrospective, single-surgeon design. Whilst operative data was prospectively recorded, long-term PRO were obtained via a non-validated telephone questionnaire, introducing the potential for recall and response bias. Despite a strong response rate (74.3%) and a median follow-up of five years, non-respondents may represent a distinct subgroup, potentially introducing selection bias. Furthermore, no objective penile length measurements were collected in the peri-operative period to corroborate subjective accounts.

## Conclusion

Both Nesbit and Yachia corporoplasty offer reliable and durable correction of uncomplicated Peyronie’s curvature, with high rates of technical success and comparable long-term patient satisfaction. Despite procedural differences, outcomes were equivalent across key domains including curvature recurrence, erectile function and altered sensation. However, subjective penile shortening was pervasive and the dominant cause of dissatisfaction, reinforcing the critical role of pre-operative counselling. These results affirm both techniques as appropriate surgical options, allowing flexibility in surgical practice and accommodating surgeon experience and preference.

## Supplementary Information

Supplementary Material 1.

## Data Availability

No datasets were generated or analysed during the current study.

## Abbreviations

PGI-I: Patient Global Impression of Improvement
PRO: Patient Reported Outcomes
