# Supplementary material for: Comparison of long-term patient reported outcomes of excisional and incisional corporoplasties for Peyronie’s disease
Source: Basic Clin Androl. 2025 Sep 9;35:36. doi: 10.1186/s12610-025-00280-3 (PMC12418631; doi:10.1186/s12610-025-00280-3)
Supplement: Supplementary file 1 — Supplementary Material 1. [file 12610_2025_280_MOESM1_ESM.docx]

# Peyronie’s ‘Patient Reported Outcomes’ Questionnaire

This questionnaire is designed to evaluate patient-reported outcomes following Peyronie’s surgery to correct penile curvature. Please answer each question to the best of your ability. The responses will remain confidential and will be used to assess treatment outcomes and improve patient care.

## A. Overall Impression and Satisfaction

**1. (PGI-I)
Prompt:** On a scale of 1 to 7, how would you rate your condition (Peyronie’s disease) now, compared to how it was before you had surgery, where

☐ 1= Very much better

☐ 2= Much better

☐ 3= A little better

☐ 4= No change

☐ 5= A little worse

☐ 6= Much worse

☐ 7= Very much worse

**2.**

**Prompt:** On a scale of 1 to 5**,** how satisfied are you overall with the results of the penile straightening surgery, where

☐ 1 = Dissatisfied

☐ 2= Somewhat dissatisfied

☐ 3= Neither satisfied nor dissatisfied

☐ 4= Somewhat satisfied

☐ 5 =Completely satisfied

**3. Prompt:** Since your penile straightening surgery, have you been able to successfully engage in penetrative sex at any stage?

☐ Yes ☐ No

**If ‘No’,**

**Prompt:** What is the main reason?

☐ I have not had a sexual partner since surgery

☐ **Sexual activity with partner has not resumed by choice or relationship circumstances**

☐ I experience ongoing sexual dysfunction related to Peyronie’s disease

☐ Other:

_________________________________________________________________________

_________________________________________________________________________

**4. Prompt:** Do you regret having the surgery to straighten your erections?

☐ Yes ☐ No

If ‘Yes’, could you briefly explain why?

_________________________________________________________________________

_________________________________________________________________________

## B. Post-Operative Outcomes

**Recurrent Curvature**

**5. Prompt:** Since your surgery, have you developed a recurrence of penile curvature?

☐ Yes ☐ No

**If ‘Yes’:**
**(a) Prompt:** **Does the current curvature interfere with penetrative sex?**

☐ Yes  ☐ No  ☐ Not applicable (not currently sexually active)

**(b) Prompt:** **Approximately how many degrees of curvature do you estimate?**

_____ degrees

**(c) Prompt:** **On a scale of 1 to 5, how bothered are you by the new curvature,** where

☐ 1 = Extremely bothered

☐ 2 = Very bothered

☐ 3 = Moderately bothered

☐ 4 = A little bothered

☐ 5 = Not at all bothered

**Penile Length**

**6. Prompt:** Since the surgery, do you believe your penis has become shorter?

☐ Yes ☐ No

**If ‘Yes’:**

**(a) Prompt:** **Approximately how many centimetres shorter do you believe it is?**
_____ cm

**(b) Prompt:** **On a scale of 1 to 5, how bothered are you by the perceived shortening, where:**
☐ 1 = Extremely bothered

☐ 2 = Very bothered

☐ 3 = Moderately bothered

☐ 4 = A little bothered

☐ 5 = Not at all bothered

**Appearance**

**7. Prompt: Thinking about the**last time**you looked at your penis, ideally while erect, how bothered were you by its appearance?**

**On a scale of 1 to 5,** where:

☐ 1 = Extremely bothered

☐ 2 = Very bothered

☐ 3 = Moderately bothered

☐ 4 = A little bothered

☐ 5 = Not at all bothered

**Sensation**

**8. Prompt: After the surgery, did you notice any numbness or decreased sensation of the penis?**☐ Yes ☐ No

**If ‘Yes’:**

**(a) Prompt: On a scale of 1 to 5, how bothered were you by the change in sensation, where:**
☐ 1 = Extremely bothered

☐ 2 = Very bothered

☐ 3 = Moderately bothered

☐ 4 = A little bothered

☐ 5 = Not at all bothered

**Erectile Function**

### 9. (a) ****Prompt:** On a scale of 1 to 5, how did the strength of your erections change following the surgery, where:**

☐ 1 = Much worse

☐ 2 = Slightly worse

☐ 3 = No different

☐ 4 = Slightly better

☐ 5 = Much better

**(b) Prompt: Have you started using any new erectile dysfunction (ED) treatments since the surgery, including any of:**

☐ Medications (e.g. Viagra, Cialis)

☐ Penile injection therapy (ICI)

☐ Vacuum erection device (VED)

☐ Inflatable penile prosthesis (IPP)

☐ No new treatment required

**Foreskin complications**

**10.** **Prompt:** Before the surgery, were you circumcised?

☐ Yes ☐ No

**If ‘No’:**
 **(a) Prompt: Were you circumcised as part of this surgery?**

☐ Yes ☐ No

**(b)**

**If ‘Yes’: Prompt:** **Did you experience any post-operative issues?**

☐ Bothersome increased sensitivity

☐ Bothersome decreased sensitivity

☐ Concerns about appearance (cosmesis)

☐ Wound healing problems

**If ‘No’: Prompt:** **Did you experience any post-operative issues?**
**Did you experience any post-operative issues?**

☐ Tight foreskin (phimosis)

☐ Persistent swelling (oedema)

☐ Decreased sensitivity

☐ Required circumcision at a later stage
